# Insights into therapeutic discovery through the Kelch domain structure of Keap1 at ambient temperature

**DOI:** 10.55730/1300-0152.2742

**Published:** 2025-04-07

**Authors:** Merve YILMAZ, Belgin SEVER, Yiğit KUTLU, Mehmet GÜL, Ceren OKUDUCU, Serra TAVLI, Masami OTSUKA, Mikako FUJITA, Türkan HALİLOĞLU, Halilibrahim ÇİFTÇİ, Hasan DEMİRCİ

**Affiliations:** 1 Department of Molecular Biology and Genetics, Faculty of Sciences, Koç University, İstanbul, Turkiye; 2 Medicinal and Biological Chemistry Science Farm Joint Research Laboratory, Faculty of Life Sciences, Kumamoto University, Kumamoto, Japan; 3 Department of Pharmaceutical Chemistry, Faculty of Pharmacy, Anadolu University, Eskişehir, Turkiye; 4 Department of Chemical Engineering, Faculty of Engineering, and Polymer Research Center, Boğaziçi University, İstanbul, Turkiye; 5 Max Delbrück Center for Molecular Medicine in the Helmholtz Association, Berlin, Germany; 6 Department of Pharmaceutical Sciences, Faculty of Pharmacy, University of Vienna, Vienna, Austria; 7 Department of Drug Discovery, Science Farm Ltd., Kumamoto, Japan; 8 Polymer Research Center, Boğaziçi University, İstanbul, Turkiye; 9 Department of Molecular Biology and Genetics, Faculty of Arts and Sciences, Burdur Mehmet Akif Ersoy University, Burdur, Turkiye; 10 Department of Engineering Sciences, Faculty of Engineering and Architecture, İzmir Katip Çelebi University, İzmir, Turkiye; 11 Koç University İşbank Center for Infectious Diseases (KUISCID), Koç University, İstanbul, Turkiye; 12 Stanford PULSE Institute, SLAC National Accelerator Laboratory, Stanford University, Menlo Park, CA, USA

**Keywords:** Keap1, Nrf2, Kelch domain, dimethyl fumarate, CNN, structural dynamics

## Abstract

**Background/aim:**

The Kelch-like-ECH associated protein 1 (Keap1) is an integral component of the E3-ubiquitin ligase complex, which binds to Nuclear factor erythroid 2-related factor 2 (Nrf2) and facilitates its degradation by the 26S proteasome. The Kelch domain of Keap1, composed of six repeated structural motifs, plays a key role in this interaction. This study aims to investigate the dimeric structure of the Keap1 Kelch domain at ambient temperature and to examine its implications for conformational dynamics, particularly in relation to the DMF and Nrf2 binding sites.

**Materials and methods:**

The dimeric crystal structure of the Keap1 Kelch domain was determined at 3.0 Å resolution using data collected at the Turkish Light Source ‘Turkish DeLight.’ To analyze structural dynamics, Gaussian Network Model (GNM) analysis was applied, and molecular docking studies were performed using the ambient temperature structure to evaluate the binding of compounds acting as inhibitors of the Keap1/Nrf2 complex.

**Results:**

The study reveals significant potential conformational changes in Keap1 residues, especially at the DMF and Nrf2 binding sites, driven by temperature-induced shifts. GNM analysis suggests that the allosteric behavior of DMF binding residues is fully realized in the ambient temperature structure. Molecular docking of various compounds, including CNN (a hybrid of L-carnosine and L-histidyl hydrazide), ZINC 12433145, and ZINC 105508677, demonstrated favorable binding interactions with key Keap1 residues, highlighting their potential as inhibitors.

**Conclusion:**

Our in silico and crystallo results suggest that CNN is a promising lead compound for Keap1 inhibition. Understanding the dimeric form of the Keap1 Kelch domain and its conformational changes at ambient temperature is crucial for elucidating the dynamics of the Keap1-Nrf2 interaction.

## 1. Introduction

A substrate adaptor protein involved in the Cullin-3-dependent E3 ubiquitin ligase complex is called Kelch-like ECH-Associated Protein 1 (Keap1). Keap1 comprises 625 amino acids and 27 cysteine residues and has a molecular weight of approximately 70 kDa. The DGR (Double Glycine Repeats)/Kelch domain of Keap1, which includes evolutionary conserved six Kelch repeats, mediates Nuclear factor erythroid 2-related factor 2 (Nrf2)-Keap1 interaction. The cryogenic structure of the human Kelch domain of Keap1 has been previously determined at 1.85 Å resolution by synchrotron X-ray crystallography. The Kelch domain is formed by assembling six repeats of bladed β-propeller structures. Kelch repeats have highly conserved glycine, tyrosine, and tryptophan residues, which have essential functional roles (Li et al., 2004). Nrf2 binds to Keap1 through the Kelch domain from two different binding motifs, ‘DLG and ETGE,’ in the Neh2 domain. The DLG (Asp-Leu-Gly) motif is found in the N-terminal region of Nrf2 and interacts with the Kelch domain. The ETGE (Glu-Thr-Gly-Glu) motif is also found in the Neh2 degron and interacts with the Kelch domain (Tong et al., 2006).

The human Keap1 is a critical part of the Keap1-Nrf2-ARE (Antioxidant Response Elements) pathway that regulates the genes involved in detoxification, cytoprotective processes, and antioxidant defense to sustain cellular homeostasis (Canning et al., 2015). Keap1 is essential in controlling the ubiquitination-mediated degradation of Nrf2, which prevents Nrf2 from translocating into the nucleus regularly. However, in response to the presence of electrophilic substances or oxidative stress, some cysteine residues in Keap1 can perturb the Keap1-Nrf2 interaction. The dissociation of Keap1 and Nrf2 allows Nrf2 to translocate to the nucleus, bind to AREs in the DNA (Deoxyribonucleic Acid), and activate the transcription of genes involved in antioxidant defense, detoxification, and cellular protection (Baird and Dinkova-Kostova, 2011).

Dimethyl fumarate (DMF) is a prodrug known to activate the Nrf2 pathway (Linker et al., 2011). In adults, it is indicated for the treatment of relapsing forms of multiple sclerosis (MS), including clinically isolated syndrome, relapsing-remitting disease, and active secondary progressive disease (Brennan et al., 2015). DMF binds to two distinct regions of Keap1: the Nrf2 binding region (Site 1) and near the second Kelch repeat (Site 2). DMF and Keap1 interaction from Site 1 is an intermediate interaction; this site is the central region of interaction between Nrf2 and Keap1 (Unni et al., 2021). These two binding sites show the DMF capability of Nrf2 function activation with covalent and noncovalent interactions. This indicates that DMF is a potential therapeutic agent when dealing with oxidative stress in the cell. In addition, DMF analogs such as monoethyl fumarate (MEF) and fumarate (FUM) also exhibit the same binding modes as DMF. The interaction of DMF analogs with Keap1 might help Nrf2 activation and deal with oxidative stress similarly (Unni et al., 2021).

Despite the significant understanding of the Keap1-Nrf2 interaction and its implications for cellular protection, the structural dynamics of Keap1 remain underexplored. Moreover, the impact of small molecules like fumarate derivatives on Keap1 conformation and Nrf2 activation, especially concerning conformational flexibility at varying temperatures, is not fully understood. This knowledge gap presents an opportunity to investigate the dimeric structure of the Keap1 Kelch domain at ambient temperature and its potential implications for drug design, particularly for compounds targeting the Keap1-Nrf2-ARE pathway.

In this study, we determined the dimeric Kelch domain of Keap1 at ambient temperature. We investigated the dimerization of the Kelch domain and identified the residues contributing to dimerization. We compared our structure with previously published structures for DMF and Nrf2 binding residues. Computationally, we performed GNM and GNM-based Transfer Entropy (GNM-TE) analysis to understand the dynamic behavior of the Kelch domain structure at cryogenic and ambient temperatures, especially the dimerization and DMF binding behaviors.

We also conducted molecular docking studies on our structure with our previously reported compound CNN (a hybrid of *L*-carnosine and *L*-histidyl hydrazide) (Noguchi et al., 2019), *L*-carnosine, carnosic acid, and fumarate derivatives. Our findings, combined with existing knowledge of the structure and function of the Kelch domain, may help to drive future research for understanding Keap1-Nrf2 dynamics and the discovery of new drugs affecting the Keap1-Nrf2-ARE pathway.

## 2. Materials and methods

### 2.1. Transformation and expression

Kelch domain of Keap1 with the codon-optimized human sequence was cloned into a pet28a plasmid, including an N-terminal hexahistidine tag with a thrombin protease cut site. As cloning restriction enzyme cut sites, NdeI and BamHI were chosen, and the kanamycin resistance gene was used as a selection marker. The constructed plasmid was transformed into a competent *Escherichia coli* (E. coli) Rosetta2™ BL21 strain as described in Ertem et al., 2022. Transformed bacterial cells were grown in 6 liters of regular Lysogeny Broth (LB) media containing 50 μg/mL kanamycin and 35 μL/mL chloramphenicol at 37 °C. When the OD_600_ value reached 1.2, the protein expression was induced using β-D-1-thiogalactopyranoside (IPTG) at a final concentration of 0.4 mM for 18 h at 18 °C. Cell harvesting was performed using a Beckman Allegra 15 R desktop centrifuge at 4 °C at 3500 rpm for 45 min. The cell pellets were stored at −80 °C until protein purification.

### 2.2. Protein purification

The cells were sonicated in a lysis buffer containing 500 mM NaCl, 20 mM Imidazole, 50 mM Tris-HCl (pH 8.5), 5% glycerol, and 0.1% Triton-X100. The cells were homogenized by dounce glass tissue homogenizer and lysed using a Branson W250 sonifier (Brookfield, CT, USA). The cell lysate was centrifuged at 4 °C at 35,000 rpm for 1 h with a Beckman Optima L-80XP Ultracentrifuge equipped with a Ti45 rotor (Beckman, Brea, CA, USA). The pellet containing membranes and cell debris was discarded. The supernatant containing the soluble protein was filtered through a 0.2-micron hydrophilic membrane and loaded to a Ni-NTA column that was previously equilibrated with an equilibrium buffer containing 200 mM NaCl, 20 mM Tris-HCl (pH 8.5), 20 mM Imidazole. The column was washed with a wash buffer to discard unbound proteins from the column. Afterward, the target protein, Keap1, was eluted using an elution buffer containing 250 mM NaCl, 20 mM Tris-HCl (pH 8.0), and 250 mM Imidazole.

### 2.3. Crystallization

The crystallization screening of Keap1 was performed using the sitting-drop microbatch under the oil method against ~3000 commercially available sparse matrix crystallization screening conditions in a 1:1 volumetric ratio in 72-Terasaki plates (Greiner Bio-One, Kremsmünster, Austria). The mixtures were covered with 16.6 μL 100% paraffin oil (Tekkim Kimya, İstanbul, Türkiye). The Terasaki plates were incubated at 4 °C and checked frequently under a stereo light microscope. The best Keap1 crystals were obtained within one month in condition Salt Rx-II #44 which contain 4.0M Ammonium Acetate, 100mM Sodium Acetate Trihydrate (pH 4.6) and Salt Rx-II #45 4.0M Ammonium Acetate, 100mM BIS TRIS Propane (pH 7.0) and Salt Rx- II #46 which contain 4.0 M Ammonium Acetate, 100mM Tris-HCl (pH 8.5) (Hampton Research, USA).

### 2.4. Ambient temperature data collection and processing

Ambient temperature X-ray crystallographic data was collected using Rigaku’s XtaLAB Synergy R Flow XRD (X-Ray Diffraction) system, as described in Gul et al., 2023. Multiple crystals were screened using the modified adapter of the XtalCheck-S plate reader. After selecting well-diffracting crystals, diffraction data were collected from two crystals. The duration of exposure time was optimized to minimize the potential radiation damage caused by X-rays. A total of 2 crystals were used to collect a complete diffraction dataset. Diffraction data were continuously collected for approximately 3 h at 90% attenuation. The detector distance was set at 120.0 mm, while the scan width was 0.5°, and the exposure time was 5.0 s per image. The diffraction data were set up in CrysAlisPro to complete the automated data collection. The collected data was then merged using the profit merge process with CrysAlisPro 1.171.42.59a software Rigaku,^1^ to produce an integrated reflection dataset (*.mtz) file for further analysis, as described in Gul et al., 2023.

### 2.5. Structure determination and refinement

The structure was determined using molecular replacement with the PHASER-MR program implemented in the PHENIX suite, using the cryogenic synchrotron Keap1 crystal structure (PDB ID: 6ROG) as the initial search model (Adams et al., 2010). Individual coordinates and TLS (Translation-Libration-Screw) parameters were refined following rigid body and simulated annealing refinement. Potential positions of altered side chains and water molecules were examined, and the models were manually constructed/reconstructed using the COOT program (Emsley and Cowtan, 2004). The final structure refinement was performed using phenix.refine in *PHENIX*. Structural figures were generated using PyMOL.

### 2.6. Gaussian network model (GNM) analysis

To analyze the structural dynamics of the Keap1 Kelch domain structures, we employed the Gaussian Network Model (GNM) (Bahar et al., 1997; Haliloglu et al., 1997). GNM decomposes the fluctuations of residues in a protein structure into a series of orthogonal modes of motion. These modes range from highly collective global motions to localized fluctuations across the protein structure. The study focuses on the slow mode spectrum, particularly the 10 and 5 slowest modes for the dimeric and monomeric structures, respectively. These normal modes generally account for a significant portion of a protein’s functional dynamics.

The equilibrium correlation between the fluctuations of two residues i and j, respectively, ΔR_i_ and ΔR_j_, is given as:


(1) $$\langle \Delta R_{i}\cdot \Delta R_{j}\rangle =(\frac{3k_{b}T}{\gamma }){\left[\Gamma^{-1}\right]}_{ij}$$

where **Γ** is a symmetric matrix of the Kirchhoff (connectivity) matrix with a r-cut of 10 Å to assume interactions, *γ* is the force constant of the Hookean pairwise potential function, representing the interactions between the residues in the folded structure. *T* is the absolute temperature in Kelvin, and *k_b_* is the Boltzmann constant. Eq. 1 can be rewritten as:


(2) $$\langle \Delta R_{i}\cdot \Delta R_{j}\rangle =\left(\frac{3k_{b}T}{\gamma }\right){\left[U(\Lambda^{-1})U^{T}\right]}_{ij}=\left(\frac{3k_{b}T}{\gamma }\right)\sum_{k=1}^{n-1}{\left[{\lambda_{k}}^{-1}u_{k}{u_{k}}^{T}\right]}_{ij}$$

where *k* is the *k*-th vibrational mode in the spectrum of *n*-1 modes, *n* being the number of residues. ***U*** is an orthogonal matrix whose columns ***u****_i_* are the eigenvectors of **Γ**, and **Λ** is the diagonal matrix of the eigenvalues *λ_k_*.

Additionally, GNM-based Transfer Entropy predictions explore protein dynamics in causal interrelation.

GNM-TE combines Transfer Entropy (TE) with GNM to assess the directional flow of information between residues i and j (Hacisuleyman & Erman, 2017; Altintel et al., 2022; Ersoy et al., 2023). TE quantifies the reduction in uncertainty about the movements of residue j given the movements of residue i, incorporating a time delay τ, represented as T_i→j_(τ). Net TE values equate to the difference between T_i-j_ (τ) and Tj-i (τ)

The TECol score for each residue, referred to as residue i, is determined by multiplying its cumulative positive net TE value (the total of positive net TE values that residue i transmits to other residues) by its collectivity value K_i,s_ within each subset s.


(3) $$TExCol\;{Score}_{i,s}=K_{i,s}\cdot \sum_{j=1}^{N}\Delta T_{i\to j,s}$$

The collectivity value K_i,s_ within each subset s is calculated as


(4) $$K_{i,s}=\frac{1}{N}exp\;\left(-\sum_{j=1}^{N}\alpha {\left(\Delta T_{i\to j,s}(\tau )\right)}^{2}log\;\left(\alpha {\left(\Delta T_{i\to j,s}(\tau )\right)}^{2}\right)\right)$$

where s represents a selected subset of slow GNM modes, N is the total number of residues, and α denotes the normalization factor. The TECol score, which combines TE and K values, is essential for identifying the most functionally significant global information source residues. These residues have substantial influence and act as powerful effectors within the protein structure.

### 2.7. P-value analysis

To demonstrate the statistical significance between functional residues (e.g., DMF binding residues) and those identified through TECol score analysis, we conducted a p-value analysis. We randomly selected the same number of residues as those identified by the TECol scores and calculated the overlap with the DMF binding residues. A DMF binding residue was considered a match if it was within 4 Å of any randomly selected residue. This process was iterated 1000 times to generate a randomly distributed population and a cumulative distribution function (CDF) for the overlaps. The p-value was then calculated using the CDF for the overlap between the DMF binding residues and those identified by the TECol scores.

### 2.8. Molecular docking studies

ZINC database^2^ (https://zinc.docking.org/) was used to obtain the proper structures for exploration of potential Keap1/Nrf2 inhibitors (fumarates and carnosic acid derivatives) and potential Nrf2 activators (*L*-carnosine and *L*-carnosine derivatives).

The crystal structure of the ambient temperature Keap1 Kelch domain, Keap1^Ambient_APO^, was acquired from the RCSB database (ref: https://www.rcsb.org/structure/8X34). The Protein Preparation Wizard was utilized to prepare the raw protein to be used in molecular docking. The missing chains were added automatically by Prime and the protonation state was calculated by PropKa at physiological pH. Afterward, the top-ranked potential receptor binding sites were identified using SiteMap. The docking grid was determined by grid generation picking the hit binding site compromising the specified residues (Asn382, Arg483, Gln530, Gly371, Phe577, Ser363, Tyr334, 525, 572, and Val369). The generated grid was used for further docking experiments.

On the other hand, compounds were sketched and cleaned in the Maestro workspace. They were prepared with energy minimization using the OPLS 2005 force field at physiological pH by the LigPrep module. Then, the best-minimized structures were submitted to the docking experiments without further modifications. The flexible ligand alignment tool was applied to superimpose our structurally similar ligands. The self-docking experiment was performed to validate the docking protocol with DMF. The optimum structure (lowest energy) was performed for the self-docking procedure. After the obtained ligand was submitted to Glide/SP docking protocols, the same docking procedures were carried out for all designed compounds (Schrödinger Release 2016-2: Schrödinger, LLC: New York, USA, Ciftci et al., 2022; Guven et al., 2023).

## 3. Results

### 3.1. The Kelch domain of Keap1 is determined in dimeric form

Here, we determined that the 62.6 kDa dimeric Kelch domain of Keap1 consists of 572 amino acids. The Keap1 crystal belongs to the P212121 orthorhombic space group with α = 90, β = 90, γ = 90, a = 75.64, b = 75.86, and c = 218.65 (Table). The dimer structure of Kelch domain Keap1 was determined to be 3 Å resolution at ambient temperature at the Turkish light source ‘Turkish Delight’ (Atalay et al., 2023). The determined dimeric Kelch domain of the Keap1 structure was deposited to the Protein Data Bank with the PDB ID:8X34 (Keap1^Ambient_APO^).

**Figure 1:**
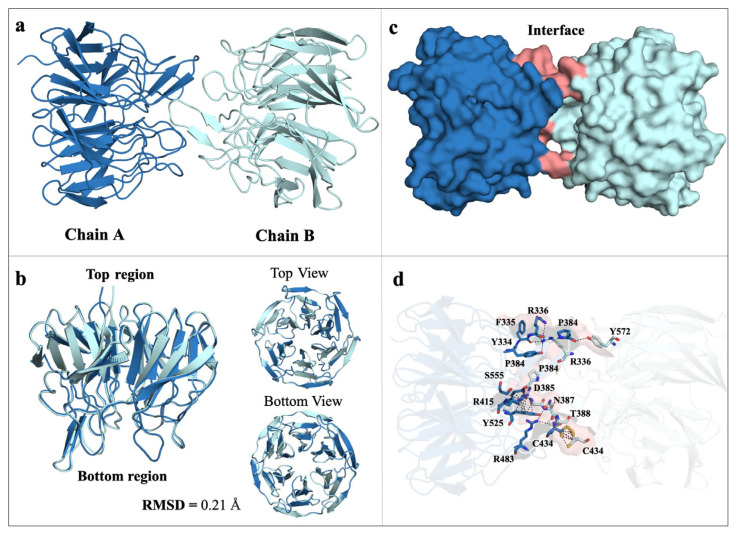
Dimerization of Kelch domain and alignment of monomers. (a) Keap1^Ambient_APO^ dimerizes through its Kelch domain, with Chain A colored in sky blue and Chain B colored in pale cyan. (b) Chain A and Chain B align with an RMSD score of 0.21 Å. The top and bottom views of Keap1 are shown. (c) The protein interface between Chain A and Chain B is shown and colored in salmon. (d) Residues contributing to the protein interface and dimerization are highlighted and shown in stick representation.

Our study suggests that the structure of Keap1 has dimerized at the Kelch domain (Figure 1a). As a result of monomer alignment, there is a slight difference between chain A and chain B, with an RMSD (Root-Mean-Square Deviation) score of 0.21 Å (Figure 1b). The residues that mediate the dimerization are shown in Figure 1c and 1d. A comparison of the dimeric Keap1^Ambient_APO^ structure at 3 Å and the dimeric Keap1Cryo_APO (PDB ID: 6ROG) structure at 2.1 Å shows a difference between the two structures with an RMSD score of 0.92 Å. To confirm that this difference results from temperature shift, the Keap1^Cryo_APO^ structure was rerefined to the resolution range of the Keap1^Ambient_APO^ (3 Å). The re-refined structure was then realigned with the PDB deposited structure. From this perspective, the resolution difference was eliminated, which suggests that the temperature shift may cause the variation. Additionally, the pairwise alignment demonstrated the distance between residues based on alpha carbons in Keap1^Ambient_APO^ and Keap1^Cryo_APO^ (Supplementary Figure S1).

### 3.2. Temperature shift causes conformational variations in DMF and NRF2 binding residues

Our structural analysis comparing the Keap1^Ambient_APO^ and cryogenic Keap1 structure in complex with DMF (Keap1^Cryo_DMF^) (PDB ID:6LRZ) structures revealed minor changes in the DMF interacting residues. These residues are found in the first Kelch repeat (Tyr334, Ser363, Val369, and Gly371), second Kelch repeat (Asn362), fourth Kelch repeat (Arg483), fifth Kelch repeat (Try525 and Glu530) and sixth Kelch repeat (Phe577 and Try572). We observed alterations on the top side Keap1 residues Asp382, Tyr334, Phe577, Tyr572, Glu530, Tyr525 and Arg483, and at the bottom side residues Val369, Gly371 (Supplementary Figure S2a). Additionally, comparing both Keap1^Ambient_APO^ and Keap1^Cryo_APO^ demonstrates similar conformational changes with the Keap1^Cryo_DMF^ structure (Supplementary Figure S2b).

We compared our Keap1^Ambient_APO^ structure with cryogenic Keap1 structure in complex with ETGE peptide (Keap1^Cryo_ETGE^) (PDB ID: 5WFN), and cryogenic Keap1 structure in complex with ETGE peptide (Keap1^Cryo_DLG^) (PDB ID: 3WN7), to determine the conformational changes in Nrf2 protein interacting residues resulting from temperature shift. Our observation suggests major alterations in the residues Arg483, Ser508, Ser555, Arg415, Ser602, Ser363, Arg380, and Asn382, which interact with ETGE and DLG peptide motifs in the ambient structure (Supplementary Figure S3).

### 3.3. GNM analysis reveals differences in residue correlations between dimeric Keap1 structures

**Figure 2:**
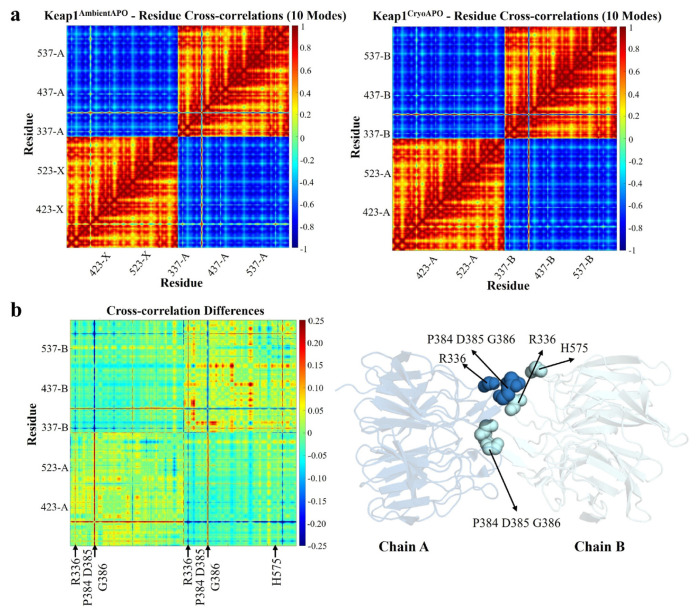
Cross-correlation maps for Keap1 apo structure in ambient and cryogenic conditions. (a) GNM residue cross-correlations for ten slowest GNM modes of the Keap1^Ambient_APO^ and at Keap1^Cryo_APO^ (b) Cross-correlation differences of these structures. Residues that show differences in correlations are marked and displayed in a 3D structure where chain A is colored in sky blue and chain B is colored in pale cyan.

**Figure 3:**
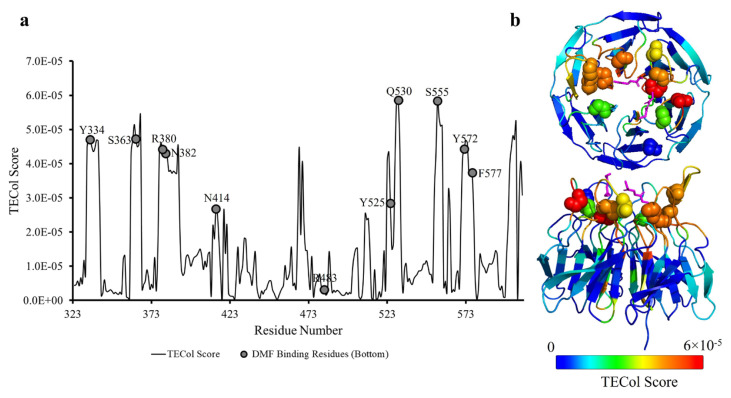
The TECol score results with the subset of slow modes consisting of GNM modes 3 to 5 for the monomer Keap1^Ambient_APO^ chain A. (a) Graphical representation of the TECol score results where bottom-side DMF binding residues are marked on the TECol score graph. (b) 3D representation of the TECol score results from two angles. Residues are colored according to the TECol score with respect to the rainbow spectrum. DMF binding residues are shown as spheres, and DMFs are shown as magenta sticks.

The structural dynamics of the Keap1 Kelch domain were investigated by examining residue cross-correlations and their differences for the ten slowest GNM modes of the Keap1^Cryo_APO^ and Keap1^Ambient_APO^ (Figure 2). The residue correlations were generally similar for both structures, with the residues predominantly correlating within their respective chains. However, residues Pro384, Asp385, and Gly386 involved in the dimerization (Figure 1) show correlated fluctuations with the other chain (Figure 2a). Additionally, we identified some variations in the correlations in specific regions, particularly near residues involved in dimerization (Figure 1), which are Arg336, Pro384, Asp385, and Gly386 for chain A, and Arg336, Pro384, Asp385, Gly386, and His575 for chain B (Figure 3). However, Arg336 and Asp385 are observed to be the residues where the β-factor between two structures is the highest, suggesting that these differences possibly occur due to thermal fluctuations (Supplementary Figure S4).

In addition, similar analyses were conducted on the monomer Keap1^Cryo_DMF^ and Keap1^Ambient_APO^ structures. The correlation profiles for these structures were very similar, and no significant differences were observed in the correlation variations (Supplementary Figure S5).

### 3.4. Ambient temperature maximizes the allosteric capacity of the DMF binding residues in the Keap1 Kelch domain

In the previous sections, it was mentioned that temperature changes cause some conformational changes in the Keap1 Kelch domain. Here, these conformational changes of the monomeric Keap1 Kelch domains were converted into a difference vector, and then the magnitude between the C-alpha atoms of each residue after superposing the monomer structures Keap1^Cryo_DMF^ and Keap1^Ambient_APO^ (chain A). Subsequently, we investigated whether a GNM mode was similar to the profile of the magnitudes of residue difference vectors. Our calculations revealed that this profile is highly correlated with the third slowest GNM mode (Supplementary Figure S6). This means that this specific dynamic mode mainly induced the conformational fluctuations from the cryogenic to ambient temperature. Although the third mode of the two structures is similar (Supplementary Figure S7), this mode may be considered significant for understanding functional dynamics.

Next, the GNM-TE method was applied to the monomeric structures Keap1^Cryo_DMF^ and Keap1^Ambient_APO^ (chain A), focusing on the five slowest GNM modes that represent the slow end of the dynamic spectrum. GNM-TE predicts the residues with the dynamic capacity to transfer information to other residues. These residues with high TECol scores are information source residues that collectively influence others and act as powerful effectors. In the five slowest GNM modes, we have used the subsets of 1 to 5, 2 to 5, and 3 to 5 slow modes. The subsets of slow modes 1 to 5 and 2 to 5 highlight the binding site residues to the molecule PG5 (see Supplementary Figure S8 for GNM-TE results of a subset of modes 1–5 and 2–5.). However, in the subset of 3 to 5 slow modes, the DMF binding regions emerged as prominent effector residues (Figure 3).

Interestingly, upon the removal of the slowest and second slowest modes, the behavior of the third slow mode, jointly with the fourth and fifth slow modes, reveals the collective behavior of the DMF binding sites, which is otherwise latent in the subset of slow modes including the two global modes. This supports the functional significance of the third slowest GNM mode we observe upon the conformational fluctuations from the cryogenic to ambient temperatures. The TECol score successfully captures DMF binding residues at or near the peak positions (<4 Å). Statistical significance analysis reveals a p-value of 0.002.

Additionally, when the same analysis was performed on the cryogenic structure, the results did not reveal the full dynamic capacity of the DMF binding sites (Supplementary Figure S9). Although there are very slight differences between these modes in the two structures (Supplementary Figure S7), it was observed that these small differences could result in variations in capturing functional movements specifically of the DMF binding regions. Furthermore, in the GNM-TE analysis for the ambient temperature structure, in addition to the DMF binding residues, Ser390, Val467, Asn469, and Gly605 also emerged as prominent effector residues. These findings imply that these residues are of allosteric importance, particularly for DMF binding behavior. The information flow graphs were also examined for residues Asn382 and Tyr334, critical for DMF binding (mentioned in the previous sections). It was observed that the information flow from these residues is very similar, and residues Tyr342, Val411, Tyr426, Ile506, Arg507, and Val514 are the ones receiving the most information transfer. These residues may also have an allosteric importance in the collective behavior induced by the DMF binding residues (Supplementary Figure S10).

### 3.5. Molecular docking studies of fumarate and carnosic acid derivatives exhibit distinct binding profiles

**Figure 4:**
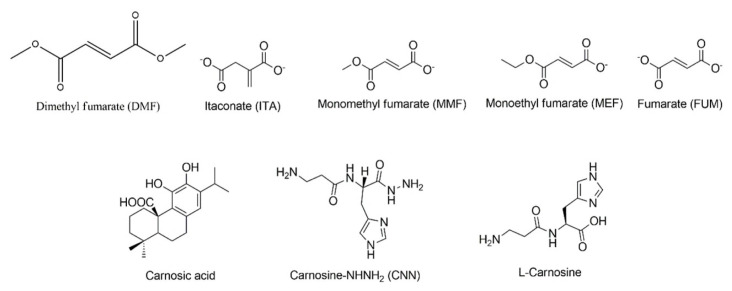
Chemical structures of L-carnosine, L-histidyl hydrazide, and their hybrid molecule CNN, alongside electrophilic fumarate derivatives (dimethyl fumarate [DMF], monomethyl fumarate [MMF], monoethyl fumarate [MEF]) and itaconate.

The molecular docking analysis of 100 fumarate derivatives in the binding site of our structure revealed that 9 derivatives (ZINC IDs: 12433145, 14496485, 3860363, 5573987, 2383091291, 21945150, 5239687, 2265312934, 1911888877) were found to possess more significant affinity profile and docking scores compared to DMF and well-known fumarates such as itaconate (ITA), monomethyl fumarate (MMF), monoethyl fumarate (MEF), and fumarate (FUM) (Figure 4).

**Figure 5:**
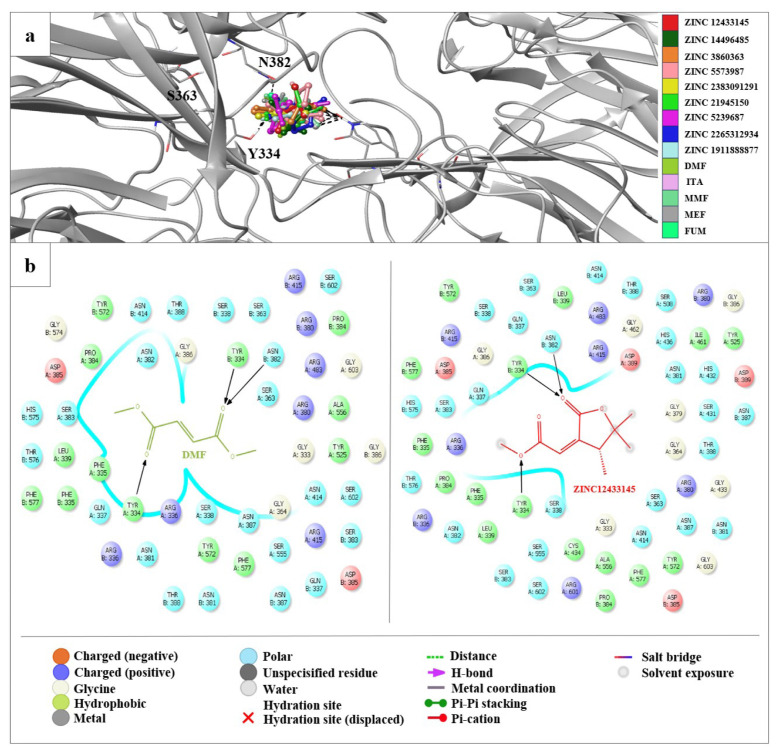
Docking results of fumarate derivatives and DMF, ITA, MMF, MEF, and FUM in the binding site of Keap1^Ambient_APO^ structure. (a) Docking poses of fumarate derivatives and DMF, ITA, MMF, MEF, and FUM in the binding site of Keap1^Ambient_APO^ structure. The light gray ribbon diagram in a metallic preset was displayed for all residues. The key residues were represented as sticks and colored by atom type: (C gray, O red, N blue). H-bonding was outlined with dotted black lines. (b) Docking interactions of ZINC 12433145 and DMF in the binding site of Keap1^Ambient_APO^ structure.

The docking score of the most effective derivative, ZINC 12433145, was detected as -9.130 kcal/mol compared to values of DMF, ITA, MMF, MEF, and FUM, which were found as −8.423, −7.533, −7.456, −7.146, and −6.934 kcal/mol, respectively. ZINC 12433145 displayed crucial hydrogen bonding with key residues Asn382 and Tyr334 in a similar pattern with DMF in the binding site of the Keap1 Kelch domain (Figure 5).

**Figure 6:**
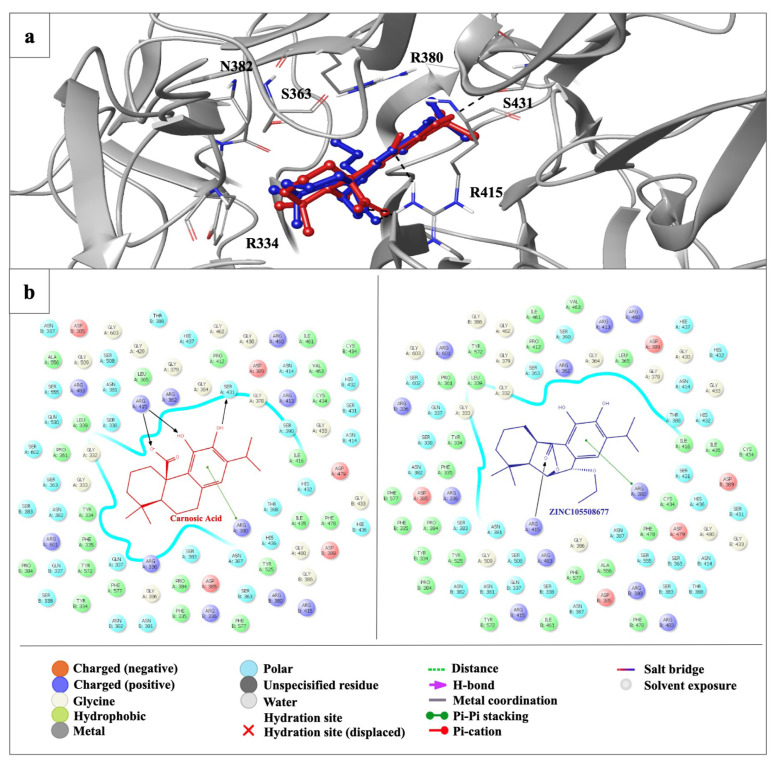
Docking results of carnosic acid and ZINC 105508677 in the binding site Keap1^Ambient_APO^ structure. (a) Docking poses of carnosic acid and ZINC 105508677 in the binding site Keap1^Ambient_APO^ structure. The light gray ribbon diagram in a metallic preset was displayed for all residues. The key residues were represented as sticks and colored by atom type: (C gray, O red, N blue). H-bond and π–π stacking interactions were outlined with dotted black and green lines, respectively. (b) Docking interactions of carnosic acid and ZINC 105508677 (colored in red and blue, respectively) in the binding site of Keap1^Ambient_APO^ structure.

The carnosic acid (Figure 4) and 100 carnosic acid derivatives exhibited a low binding profile compared with fumarate derivatives in the binding site of the Keap1 Kelch domain. Carnosic acid was identified as the most significant, with a docking score of −5.446 kcal/mol compared to the most promising carnosic acid derivative, ZINC 105508677 (−4.758 kcal/mol). Carnosic acid and ZINC 105508677 formed hydrogen bonding and π stacking with mainly arginine residues, indicating a distinct binding profile than fumarates (Figures 6a and 6b).

### 3.6. Molecular docking studies suggest CNN as the most promising drug candidate compared to L-carnosine derivatives

**Figure 7:**
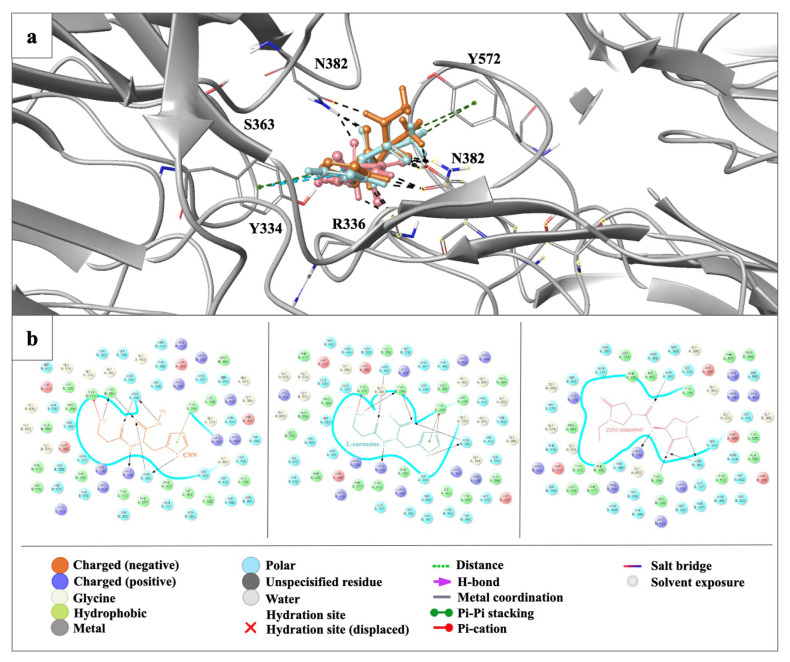
Docking results of L-carnosine, CNN, and ZINC 450609947 in the binding site of Keap1^Ambient_APO^ structure. (a) Docking poses of L-carnosine, CNN, and ZINC 450609947 in the binding site of Keap1^Ambient_APO^ structure. The light gray ribbon diagram in a metallic preset was displayed for all residues. The key residues were represented as sticks and colored by atom type: (C gray, O red, N blue). H-bond, π–π stacking, and π cation interactions were outlined with dotted black, green, and blue lines. (b) Docking interactions of CNN, L-carnosine, and ZINC 450609947 (colored in orange, turquoise, and pink, respectively) in the binding site of Keap1^Ambient_APO^ structure.

We obtained the most significant molecular docking results with our previously synthesized compound, CNN (Figure 4) (Noguchi et al., 2019). As CNN is an antioxidant *L*-carnosine derivative, we also performed in silico studies for *L*-carnosine (Figure 4) and 100 *L*-carnosine derivatives. According to the results, CNN demonstrated the most promising results in the current study. The docking score of CNN was observed as -11.532 kcal/mol, followed by the values of the most promising *L*-carnosine derivative ZINC 450609947 (−10.529 kcal/mol) and *L*-carnosine (−9.835 kcal/mol). CNN formed strong hydrogen bonding and π cation with Arg336, Asn382, Tyr334, and Tyr572 at the ionized state. The imidazole ring and carbonyl groups greatly contributed to the high affinity of CNN and *L*-carnosine. In contrast, ZINC 450609947 presented a key hydrogen bonding through its pyrazolidinone and carboxamido moieties (Figures 7a and 7b).

## 4. Discussion

In this study, our 3Å ambient temperature structure provides details of the dimeric form Keap1 Kelch domain. Our results suggest that the Keap1 Kelch domain dimerizes, with a slight difference between chains A and B (RMSD score of 0.21 Å). Previous studies have shown the monomeric structure of the Kelch domain at cryogenic temperatures. In the Protein Data Bank, only one structure of the dimeric Kelch domain has been released, Keap1^Cryo_APO^. However, the Kelch domain dimerization has yet to be investigated. A comparative analysis of the Keap1^Ambient_APO^ and Keap1^Cryo_APO^ structures showed an RMSD score of 0.92 Å, associated with temperature-based conformational changes. Specific residues involved in dimerization, such as P384, D385, and G386, exhibited correlated fluctuations across chains.

Previous studies have shown that full-length Keap1 exists in a dimeric form, with dimerization occurring through the BTB domain, and that the distance between the Kelch domains may be critical for the Keap1-Nrf2 interaction (Ogura et al., 2010). The occurrence of the dimeric Kelch domain requires further investigation to elucidate the enthalpy-entropy-based binding dynamics of the Keap1-Nrf2 interaction.

In this work, we performed GNM and GNM-TE analysis to explore the dynamic behavior of the Kelch domain structure at ambient and cryogenic temperatures, particularly for the dimerization and DMF binding behaviors. The GNM-TE method quantitatively assesses the sources and receivers of allosteric signals within a protein. This approach pinpoints the residues that serve as the most functionally significant sources of global information. These key residues exert considerable influence and function as potent effectors in the protein’s structure (Hacisuleyman and Erman, 2017; Altintel et al., 2022; Ersoy et al., 2023).

When examining the residue correlations in the dimer structures, differences were observed in the residues, possibly affecting the dimerization. On the other hand, these residues also correspond to regions where the β-factor values between the Keap1^Ambient_APO^ and Keap1^Cryo_APO^ differ the most, implying that these differences may arise from thermal fluctuations.

Our study revealed alterations in key residues such as Tyr334, Ser363, Val369, Gly371, Arg483, Tyr525, Glu530, Tyr572, and Phe577 when comparing the Keap1^Ambient_APO^ with Keap1^Cryo_DMF^ and Keap1^Cryo_APO^. Specifically, we observed that residues on the top side of Keap1, including Asp382, Tyr334, Phe577, Tyr572, Glu530, Tyr525, and Arg483, and those on the bottom side, such as Val369 and Gly371, exhibited significant conformational shifts. These variations were not only observed in the DMF binding residues but also in those involved in Nrf2 interaction, including Arg483, Ser508, Ser555, Arg415, Ser602, Ser363, Arg380, and Asn382, when comparing the Keap1^Ambient_APO^ structure with Keap1^Cryo_ETGE^ and Keap1^Cryo_DLG^. The temperature-based conformational shifts in these residues may influence the binding dynamics and stability of Nrf2.

Additionally, the GNM-TE analysis displays that the allosteric potential of the DMF binding residues was better revealed on the Keap1^Ambient_APO^ structure than the Keap1^Cryo_DMF^. In this analysis, within the subset of modes where DMF binding residues are identified as entropy sources, Ser390, Val467, Asn469, and Gly605 were also recognized as significant effector residues. These residues are proposed to have potential allosteric importance in DMF binding behavior.

Most compounds effective on the Nrf2 pathway possess electrophilic characteristics. These compounds bind to Keap1 and enable Nrf2 to translocate from the cytoplasm into the nucleus. They can be exemplified as follows: fumarates acting as Michael acceptors with an enone moiety or catechol-type compounds such as carnosic acid, which can acquire electrophilic potential by diverse mechanisms (Satoh et al., 2008; Lu et al., 2016). These electrophiles are irreversible indirect inhibitors of the Keap1/Nrf2 complex. This unspecific binding might cause less modulatory effects on the target and several adverse effects. Some potent direct noncovalent inhibitors of the Keap1/Nrf2 interactions were discovered based on the fact that the Kelch binding pocket is quite polar and basic and capable of interacting with carboxylic acid residues in both the ETGE and DLG motifs. Until now, various compounds carrying tetrahydroisoquinoline, 1,4-diaminonaphthalene, pyrazole, thiazole, indole, and triazole cores have been determined as potent Keap1-Nrf2 inhibitors. The carboxylic acid moiety was commonly introduced to the main structure for high efficacy with the basic Kelch binding pocket in these small molecules. However, the poor blood−brain barrier (BBB) permeability and propensity for conjugation reactions leading to reactive and toxic metabolites of carboxylic acids have shifted this approach into the replacement of the acidic groups with carboxamides and other bioisosteres (Pallesen et al.,2018; Ontoria et al., 2020; Sun et al., 2022; Barreca et al., 2023).

L-carnosine is an endogenous antioxidant and radical scavenger dipeptide composed of *β*-alanine and *L*-histidine. It possesses metal chelating, antiinflammatory, and neuroprotective properties, making it suitable for investigating disorders such as metabolic, cardiovascular, and neurodegenerative diseases. The underlying mechanism of antiinflammatory, antioxidant, antiglycation, and anticarbonyl effects of *L*-carnosine was mainly attributed to the activation of the Nrf2 pathway. However, the exact connection between carnosine and Nrf2 expression is still unclear (Aldini et al., 2021; Zhou et al., 2021; Caruso et al., 2022; 2023). The effects of *L*-carnosine on the Nrf2 pathway could be relevant to its interactions with Keap1. In previous work, our research group synthesized the hybrid of *L*-carnosine and *L*-histidyl hydrazide (CNN). We previously reported that CNN displayed significant 4-hydroxy-trans-2-nonenal (4-HNE) scavenging activity and diminished delayed neuronal death in the hippocampus (Noguchi et al., 2019).

We also performed molecular docking studies for different fumarate derivatives, *L*-carnosine, CNN, and other L-carnosine derivatives, and carnosic acid and its derivatives in the Kelch domain of Keap1. CNN achieved the most promising docking results in the Keap1 Kelch domain. It formed strong interactions with key residues via both *L*-carnosine and L-histidyl hydrazide parts, which also interprets the high binding potential of *L*-carnosine. Fumarate derivatives bind to the Kelch domain more effectively than well-known fumarates such as DMF, ITA, MMF, MEF, and FUM. In particular, the outcome of the molecular docking of ZINC 12433145 in the Kelch domain indicated that the conversion of one of the alkyl esters to a lactone (cyclic ester) could increase the affinity of fumarate derivatives. Although ZINC 12433145 and DMF revealed similar binding profile patterns, the distances of hydrogen bonding between ZINC 12433145 and Tyr334 were found to be greater than that of DMF, which increased occupation of the whole ligand in the Kelch domain, leading to higher affinity. On the other hand, carnosic acid and its derivatives showed less binding efficacy than all tested compounds despite their prominent carboxylic acid and/or ester groups, respectively.

In conclusion, our study comprehensively analyzes the dimeric Keap1 Kelch domain, emphasizing the differences in structural dynamics between ambient and cryogenic temperatures and their implications for Keap1-Nrf2 interactions. The observed temperature-based conformational shifts, particularly in key residues involved in dimerization as well as Nrf2 and DMF binding, suggest potential inferences for the binding dynamics and stability of the Keap1-Nrf2 complex. The GNM and GNM-TE analyses uncover crucial residues contributing to the allosteric effects and binding behaviors of various compounds. Remarkably, CNN, a hybrid of *L*-carnosine and *L*-histidyl hydrazide, demonstrates higher binding affinity and potential as an effective inhibitor compared to fumarate and carnosic acid derivatives. Our findings underline the importance of further exploring the enthalpy-entropy-based dynamics of Keap1 to better understand its interaction with Nrf2 and to guide the development of more effective therapeutic agents targeting the Keap1-Nrf2 pathway.

Despite the significant insights provided by this study, several limitations should be addressed in future research. These include validating our findings through in vitro and in vivo studies, particularly regarding the interaction of compounds like CNN, ZINC12433145, and ZINC105508677 with the Keap1 Kelch domain. Although molecular docking simulations have provided initial insights, obtaining cocrystal structures of these compounds bound to the Kelch domain would provide definitive experimental evidence of their binding modes. Furthermore, while this study has contributed to elucidating the dimerization of the Kelch domain, further research is required to investigate the dimerization of the full-length Keap1 protein and its implications for Keap1-Nrf2 interaction dynamics and functional modulation.

## Supplementary information

Supplementary Figure S1 Superposition of 2.16 Å Keap1^Cryo_APO^ (colored in gray50) and 3 Å Keap1^Ambient_APO^ (colored in sky blue and pale cyan according to monomers), with an RMSD score of 0.92 Å. Superposition of 2.16 Å Keap1^Cryo_APO^ and 3 Å Keap1^Cryo_APO^ (colored in wheat), with an RMSD score of 0.09 Å. Alignment of 2.16 Å Keap1^Cryo_APO^ and Keap1^Ambient_APO^ Chain A with an RMSD score of 0.34 Å, demonstrated by a pairwise distance plot. Alignment of 2.16 Å Keap1^Cryo_APO^ and Keap1^Ambient_APO^ Chain B with an RMSD score of 0.31 Å, demonstrated by a pairwise distance plot.

Supplementary Figure S2 (a) Superposition of Keap1^Ambient_APO^ and Keap1^Cryo_DMF^ (colored in gray80) is shown both from the bottom and top views, with DMF binding residues displayed in stick representation. (b) Keap1^Ambient_APO^ and Keap1^Cryo_APO^ (colored in gray50) are superposed, with DMF interaction residues also shown in stick representation. DMF interaction residues are displayed with their respective electron density.

Supplementary Figure S3 (a) Superposition of Keap1^Ambient_APO^ and Keap1^Cryo_ETGE^ (colored in white), with interacting residues displayed in stick representation. Superposition of Keap1^Ambient_APO^ and Keap1^Cryo_DLG^ (colored in white), with interacting residues displayed in stick representation. (b) Superposition of Keap1^Ambient_APO^ and Keap1^Cryo_APO^ (colored in gray50), with ETGE and DLG interacting residues also shown in stick representation. NRF2 interacting residues are displayed with their respective electron density.

Supplementary Figure S4 β-factor differences between the two dimeric structures obtained at ambient (Keap1^Ambient_APO^) and cryogenic (Keap1^Cryo_APO^) temperatures.

Supplementary Figure S5 GNM residue cross-correlations for the five slowest GNM modes of the monomer Keap1 Kelch domain structures Keap1^Ambient_APO^ (Chain A) and the Keap1^Cryo_DMF^ (Monomer), together with the differences of the residue cross-correlations. Only two residues (N469 & I559) show slight differences in correlations.

Supplementary Figure S6 Mean squared residue fluctuations of the third slowest mode of the kelch domain of Keap1^Cryo_DMF^ and the difference vector between Keap1^Cryo_DMF^ and Keap1^Ambient_APO^ (Chain A), together with the correlation coefficient between each of the five slowest GNM modes and the difference vector.

Supplementary Figure S7 Mean squared residue fluctuations of the five slowest GNM modes of the Keap1 Kelch domain structures obtained at cryogenic (Keap1^Cryo_DMF^) and ambient (Keap1^Ambient_APO^ Chain A) temperature. Correlation coefficients between slow modes are displayed at the top right corner of the graphs.

Supplementary Figure S8 The TECol score results with a subset of slow modes consisting of GNM modes 1 to 5 (a) and 2 to 5 (b) for the monomer Keap1 Kelch domain obtained at ambient temperature (Keap1^Ambient_APO^ chain A). DMF binding and PG5 binding residues are marked on the graphs. 3D representation of the TECol score results are given from two angles. Residues are colored according to the TECol score with respect to the rainbow spectrum. PG5 binding residues are shown as spheres, and PG5 as magenta spheres.

Supplementary Figure S9 The TECol score results with a subset of slow modes consisting of GNM modes 3 to 5 for the monomer Keap1 Kelch domain obtained at cryogenic temperature (Keap1^Cryo_DMF^).

Supplementary Figure S10 (a) 2D graph of the information flow from residues Y334 and N382 obtained from GNM-TE analysis with the subset of slow modes consisting of GNM modes 3 to 5 for the monomer Keap1 Kelch domain obtained at ambient temperature (Keap1^Ambient_APO^ chain A). (b) 3D representation of the information flow from residue Y334, where the residues are colored according to Net TE values with respect to the rainbow spectrum. The residues that receive the highest information (Y342, V411, Y426, I506, R507, and V514) are represented as spheres, and residues Y334 and N382 are represented as magenta spheres.

## Figures and Tables

**Table:** Data collection and refinement statistics.

Dataset	KEAP1
Wavelength (Å)	3.00 Å
Resolution range	31.15–3.0 (3.107–3.0)
Space group	P 21 21 21
Unit cell	a = 75.644 Å b = 75.825 Å c = 218.656 Å α = 90° β = 90° γ = 90°
Unique reflections	25889 (2543)
Completeness (%)	99.76 (98.72)
Mean I/sigma(I)	2.3
Wilson B-factor	58.83
R-merge	2.582
R-meas	2.606
R-pim	0.347
CC1/2	0.763
CC *	0.930
Reflections used in refinement	23737 (2543)
Reflections used for R-free	2162 (204)
R-work	0.2754 (0.3987)
R-free	0.3291 (0.4443)
CC (work)	0.185
CC (free)	0.042
Number of nonhydrogen atoms	4441
Proteins	4362
Ligands	0
Solvent	54
Protein residues	572
RMS(bonds)	0.010
RMS(angles)	1.36
Ramachandran favored (%)	91.90
Ramachandran allowed (%)	7.57
Ramachandran outliers (%)	0.53
Rotamer outliers (%)	7.56
Clashscore	22.36
Average B-factor	62.62
Macromolecules	62.98
Solvent	32.95
Number of TLS groups	14

## Footnotes

1 Rigaku (2022). CrysAlisPro software system, version 1.171.42.59a [online]. Website: https://www.rigaku.com/ [accessed 10 January 2024]

2 ZINC Database (2012). Irwin, Sterling, Mysinger, Bolstad & Coleman. ZINC – A Free Database of Commercially-Available Compounds for Virtual Screening. Journal of Chemical Information and Modeling DOI: 10.1021/ci3001277 [online]. Website: https://zinc.docking.org/ [accessed 8 April 2024]
