# Supplementary figures and images for: Insights into therapeutic discovery through the Kelch domain structure of Keap1 at ambient temperature
Source: Turk J Biol. 2025 Apr 7;49(3):247–60. doi: 10.55730/1300-0152.2742 (PMC12266349; doi:10.55730/1300-0152.2742)

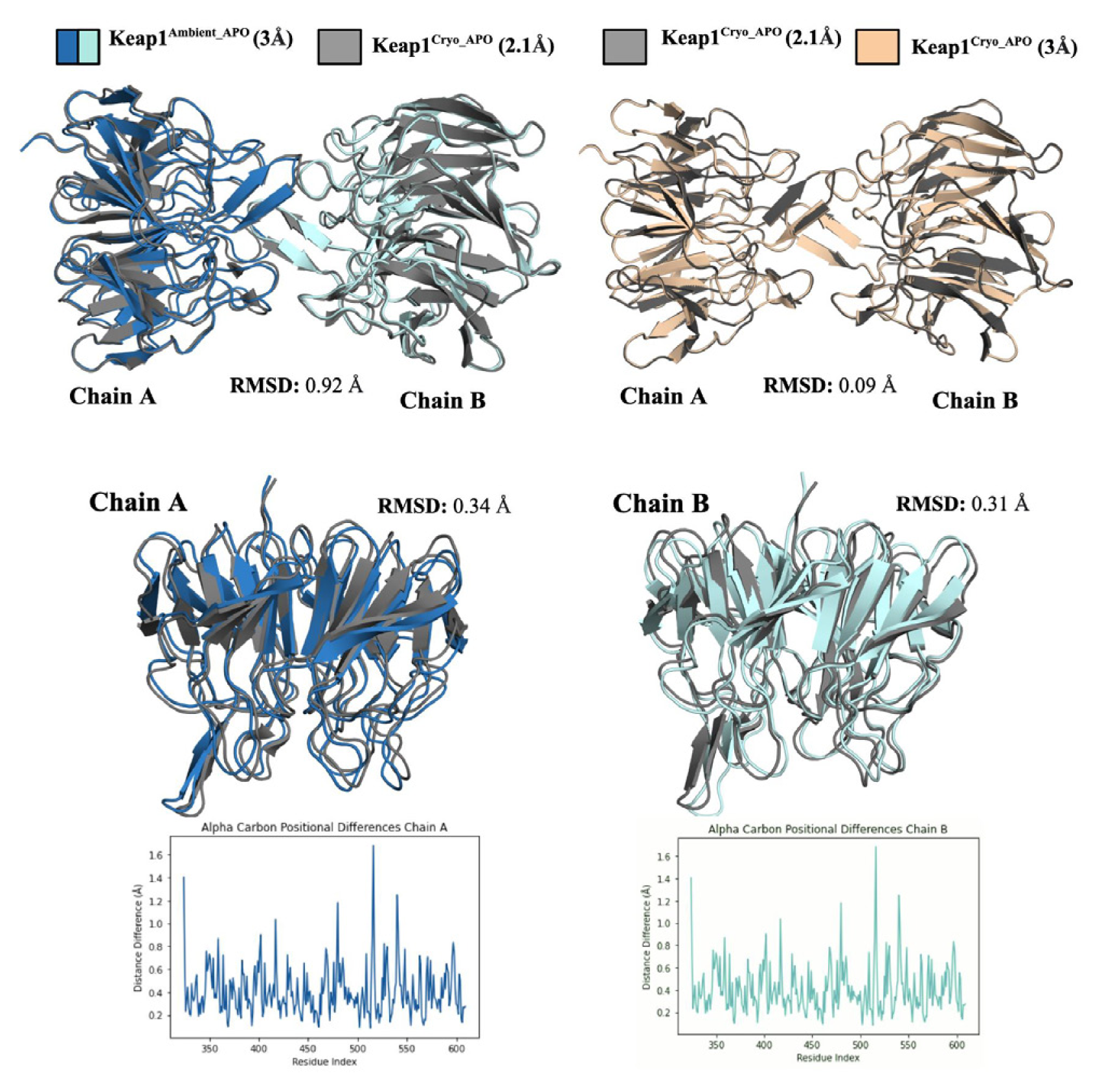

Supplement: Supplementary Figure S1 — Superposition of 2.16 Å Keap1Cryo_APO (colored in gray50) and 3 Å Keap1Ambient_APO (colored in sky blue and pale cyan according to monomers), with an RMSD score of 0.92 Å. Superposition of 2.16 Å Keap1Cryo_APO and 3 Å Keap1Cryo_APO (colored in wheat), with an RMSD score of 0.09 Å. Alignment of 2.16 Å Keap1Cryo_APO and Keap1Ambient_APO Chain A with an RMSD score of 0.34 Å, demonstrated by a pairwise distance plot. Alignment of 2.16 Å Keap1Cryo_APO and Keap1Ambient_APO Chain B with an RMSD score of 0.31 Å, demonstrated by a pairwise distance plot. [file tjb-49-03-247s1.tif]

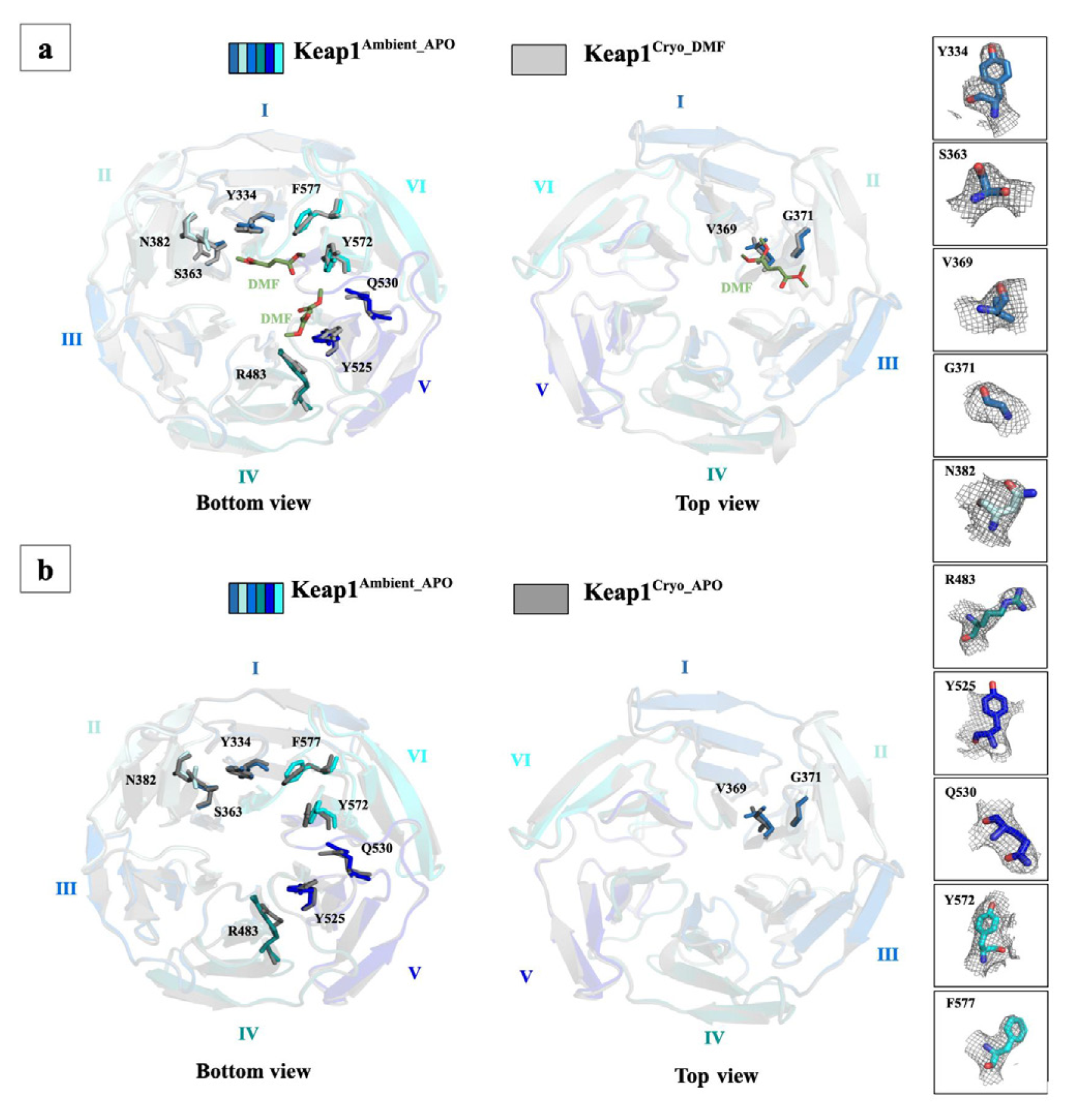

Supplement: Supplementary Figure S2 — (a) Superposition of Keap1Ambient_APO and Keap1Cryo_DMF (colored in gray80) is shown both from the bottom and top views, with DMF binding residues displayed in stick representation. (b) Keap1Ambient_APO and Keap1Cryo_APO (colored in gray50) are superposed, with DMF interaction residues also shown in stick representation. DMF interaction residues are displayed with their respective electron density. [file tjb-49-03-247s2.tif]

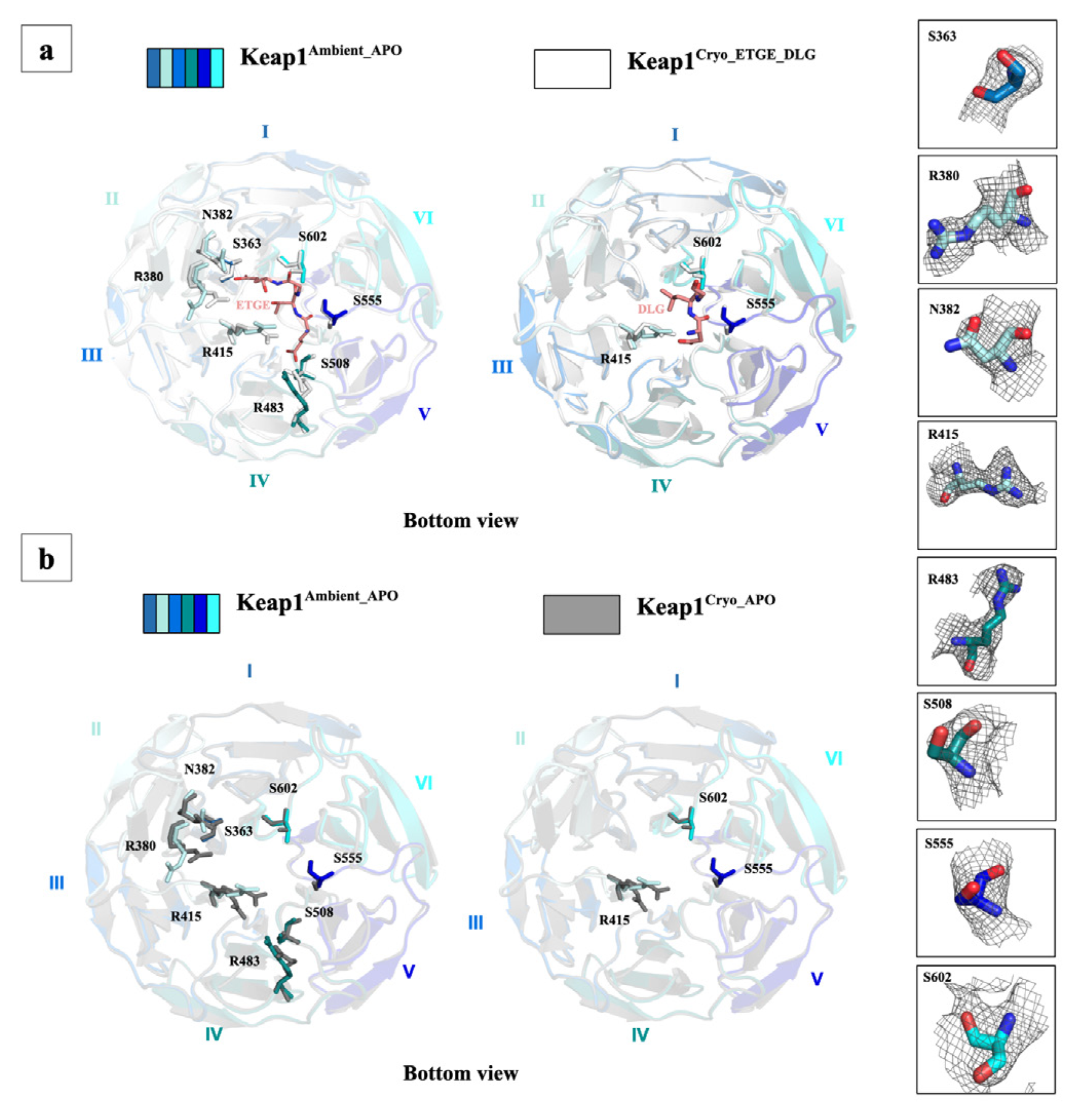

Supplement: Supplementary Figure S3 — (a) Superposition of Keap1Ambient_APO and Keap1Cryo_ETGE (colored in white), with interacting residues displayed in stick representation. Superposition of Keap1Ambient_APO and Keap1Cryo_DLG (colored in white), with interacting residues displayed in stick representation. (b) Superposition of Keap1Ambient_APO and Keap1Cryo_APO (colored in gray50), with ETGE and DLG interacting residues also shown in stick representation. NRF2 interacting residues are displayed with their respective electron density. [file tjb-49-03-247s3.tif]

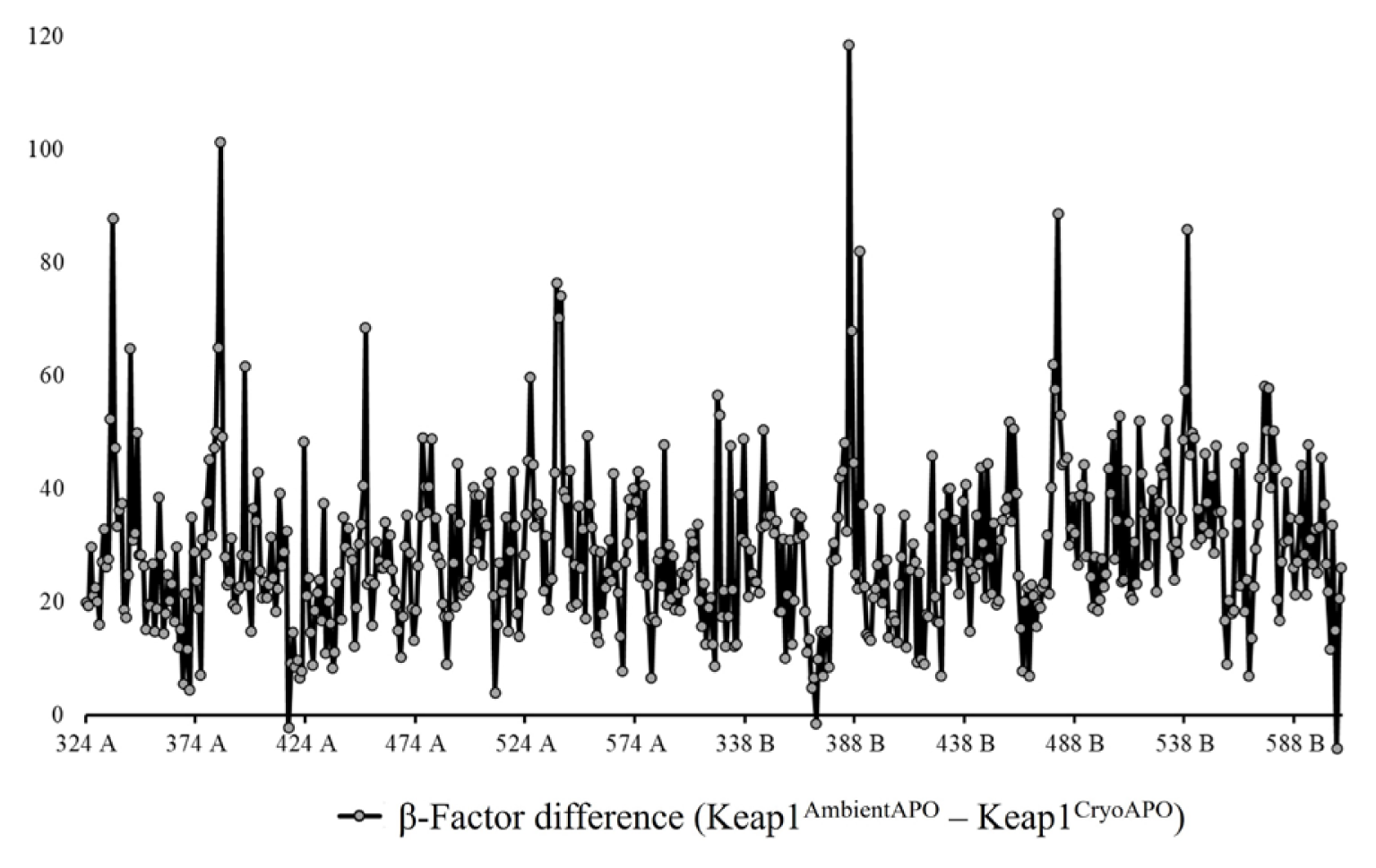

Supplement: Supplementary Figure S4 — β-factor differences between the two dimeric structures obtained at ambient (Keap1Ambient_APO) and cryogenic (Keap1Cryo_APO) temperatures. [file tjb-49-03-247s4.tif]

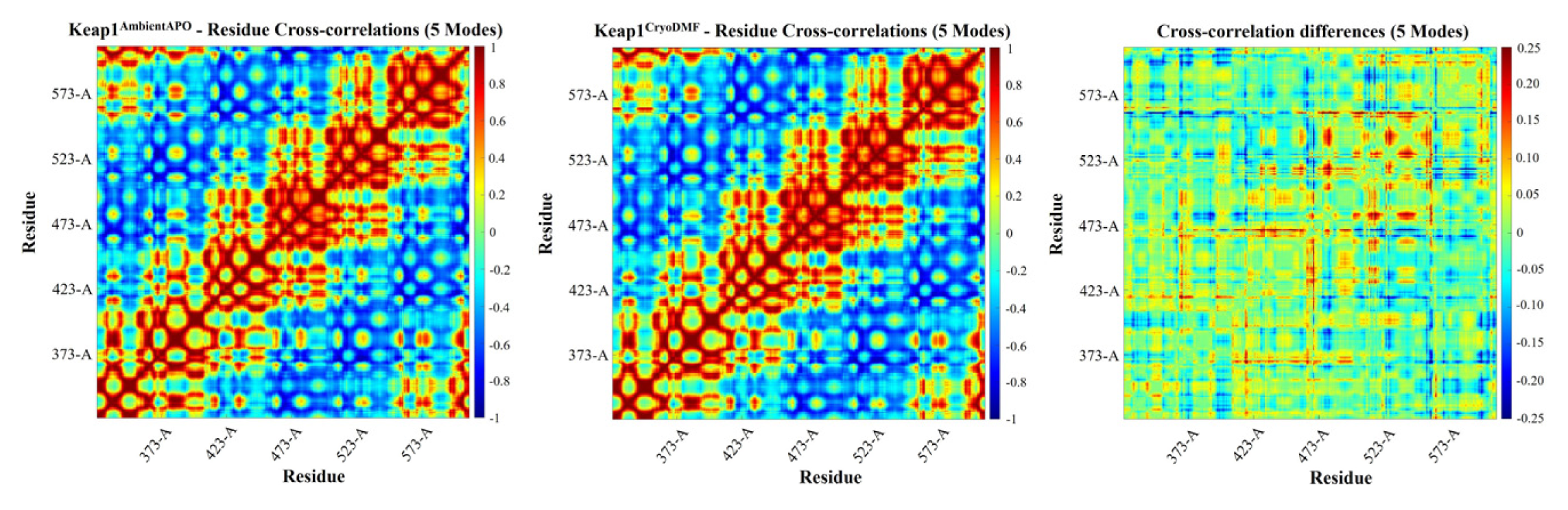

Supplement: Supplementary Figure S5 — GNM residue cross-correlations for the five slowest GNM modes of the monomer Keap1 Kelch domain structures Keap1Ambient_APO (Chain A) and the Keap1Cryo_DMF (Monomer), together with the differences of the residue cross-correlations. Only two residues (N469 & I559) show slight differences in correlations. [file tjb-49-03-247s5.tif]

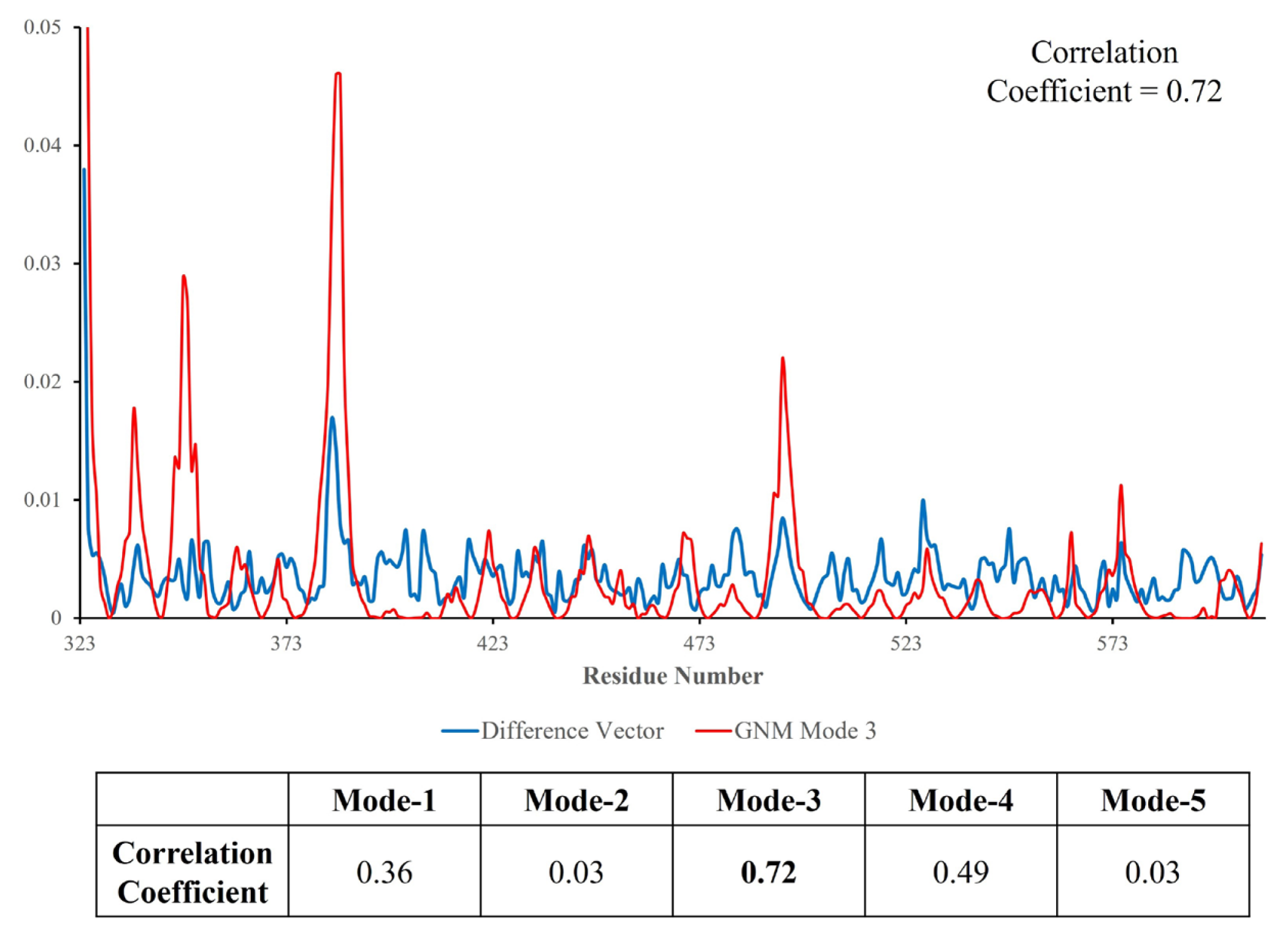

Supplement: Supplementary Figure S6 — Mean squared residue fluctuations of the third slowest mode of the kelch domain of Keap1Cryo_DMF and the difference vector between Keap1Cryo_DMF and Keap1Ambient_APO (Chain A), together with the correlation coefficient between each of the five slowest GNM modes and the difference vector. [file tjb-49-03-247s6.tif]

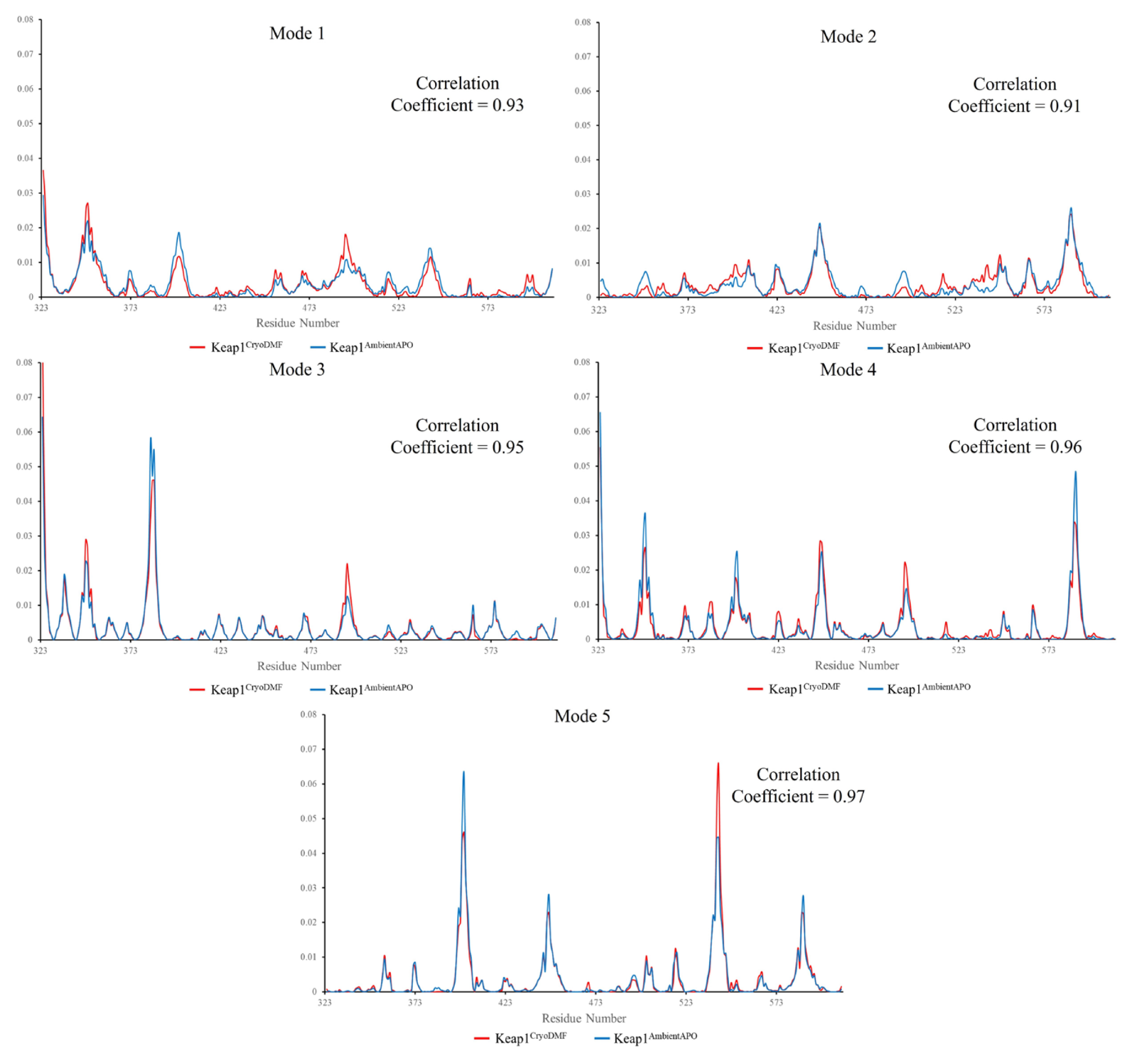

Supplement: Supplementary Figure S7 — Mean squared residue fluctuations of the five slowest GNM modes of the Keap1 Kelch domain structures obtained at cryogenic (Keap1Cryo_DMF) and ambient (Keap1Ambient_APO Chain A) temperature. Correlation coefficients between slow modes are displayed at the top right corner of the graphs. [file tjb-49-03-247s7.tif]

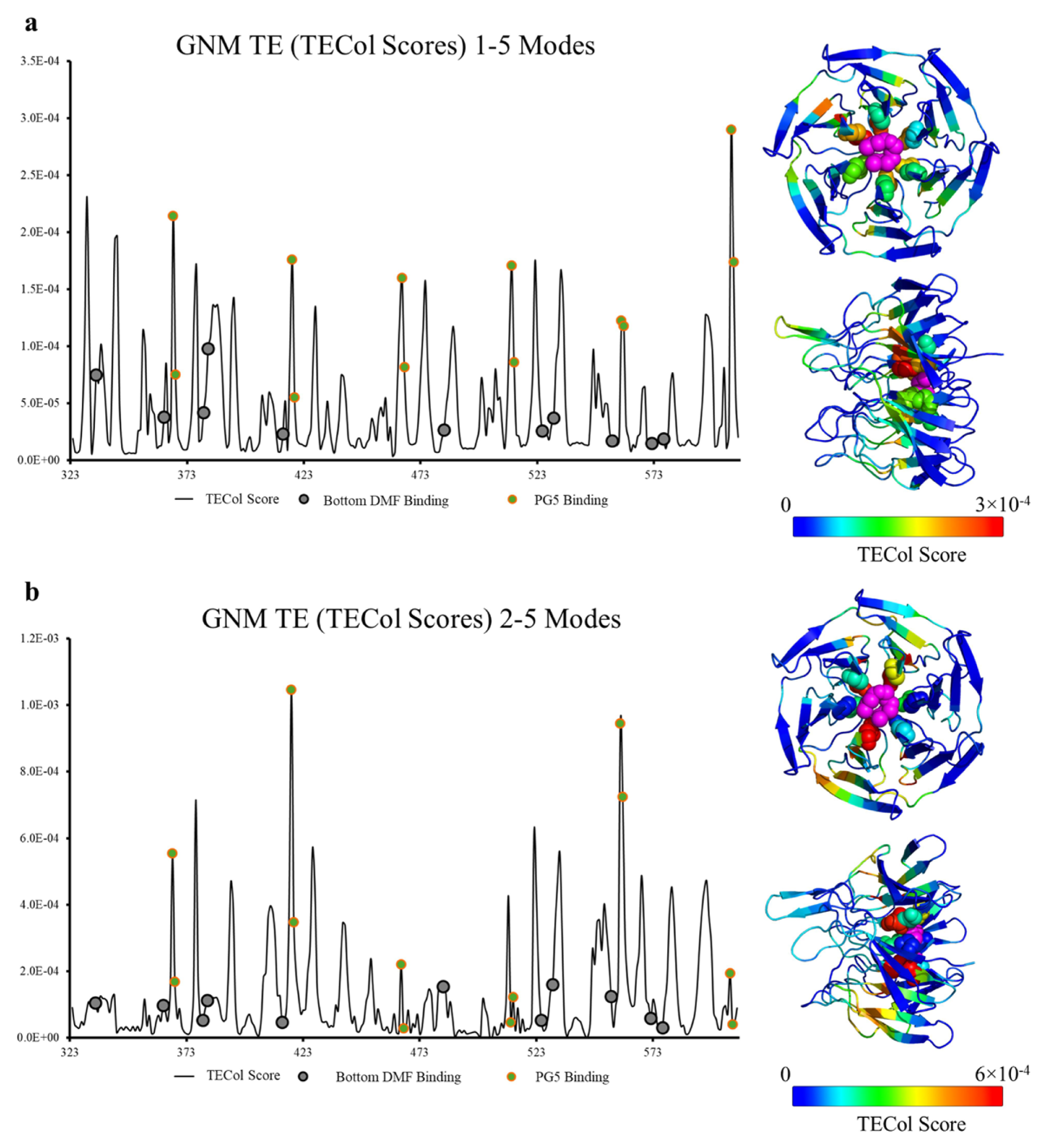

Supplement: Supplementary Figure S8 — The TECol score results with a subset of slow modes consisting of GNM modes 1 to 5 (a) and 2 to 5 (b) for the monomer Keap1 Kelch domain obtained at ambient temperature (Keap1Ambient_APO chain A). DMF binding and PG5 binding residues are marked on the graphs. 3D representation of the TECol score results are given from two angles. Residues are colored according to the TECol score with respect to the rainbow spectrum. PG5 binding residues are shown as spheres, and PG5 as magenta spheres. [file tjb-49-03-247s8.tif]

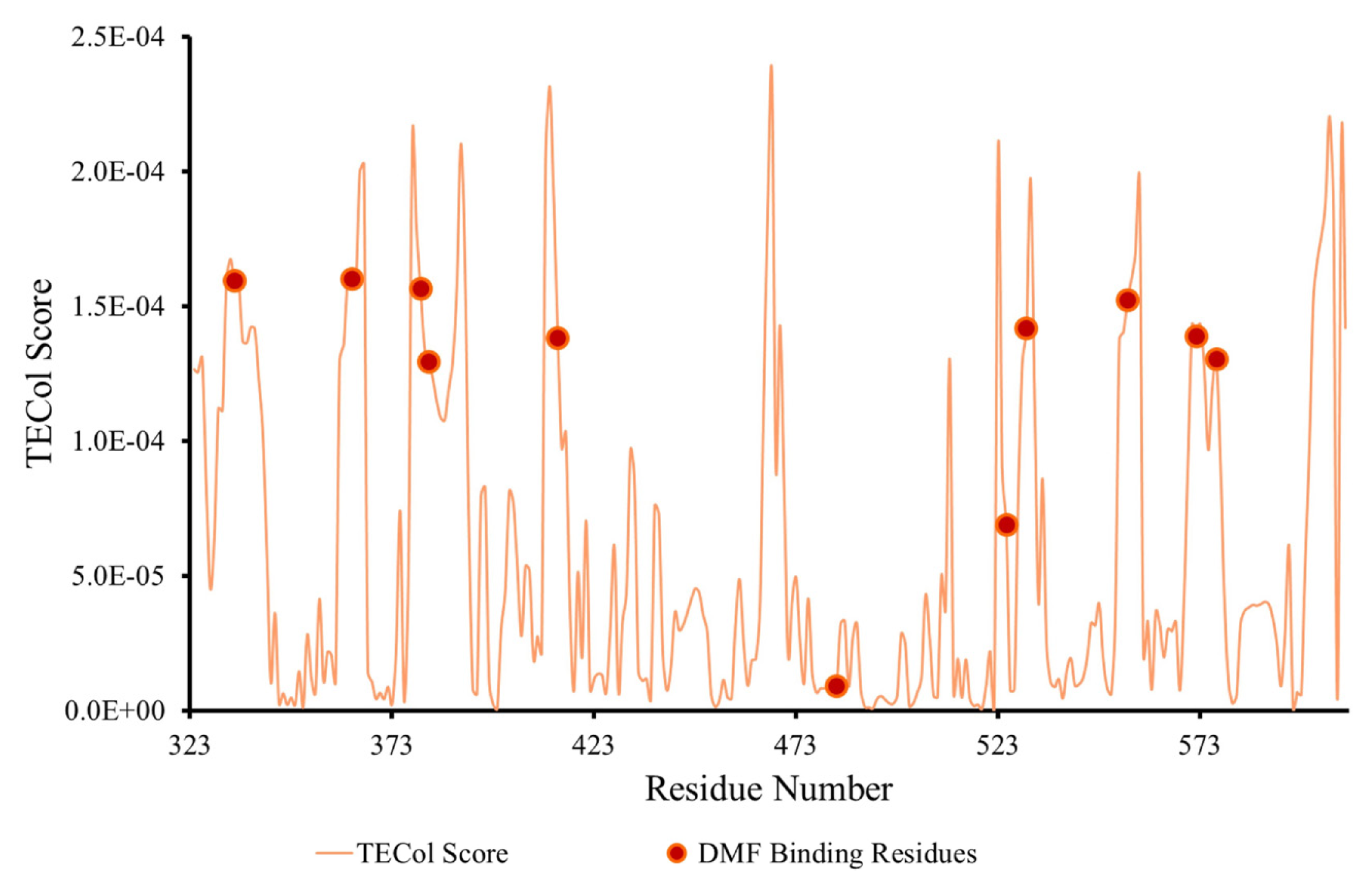

Supplement: Supplementary Figure S9 — The TECol score results with a subset of slow modes consisting of GNM modes 3 to 5 for the monomer Keap1 Kelch domain obtained at cryogenic temperature (Keap1Cryo_DMF). [file tjb-49-03-247s9.tif]

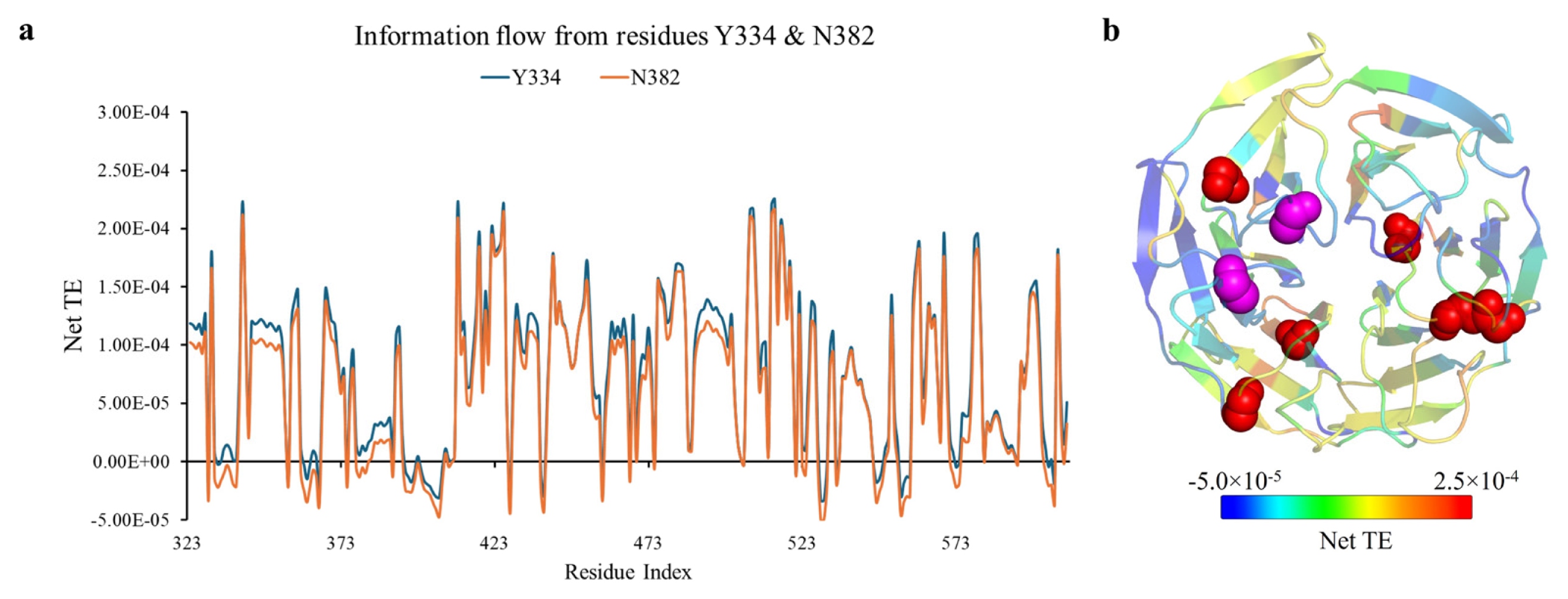

Supplement: Supplementary Figure S10 — (a) 2D graph of the information flow from residues Y334 and N382 obtained from GNM-TE analysis with the subset of slow modes consisting of GNM modes 3 to 5 for the monomer Keap1 Kelch domain obtained at ambient temperature (Keap1Ambient_APO chain A). (b) 3D representation of the information flow from residue Y334, where the residues are colored according to Net TE values with respect to the rainbow spectrum. The residues that receive the highest information (Y342, V411, Y426, I506, R507, and V514) are represented as spheres, and residues Y334 and N382 are represented as magenta spheres. [file tjb-49-03-247s10.tif]
